# Short-term safety and effectiveness of the mCLIP partial prosthesis

**DOI:** 10.1007/s00405-023-08359-1

**Published:** 2023-12-22

**Authors:** Thomas Rasse, Lisa Niederwanger, Joachim Hornung, Lava Taha, Susan Arndt, Christian Offergeld, Dirk Beutner, Nicholas Bevis, Thomas Lenarz, Magnus Teschner, Esther Schimanski, Georg Sprinzl, Astrid Magele, Piotr H. Skarżyński, Łukasz Plichta, Christoph Arnoldner, Dominik Riss, Benjamin Loader, Franz Windisch, Nina Rubicz, Paul Martin Zwittag

**Affiliations:** 1 Department of Otorhinolaryngology, Klinikum Wels-Grieskirchen, 4600 Wels, Austria; 2 Department of Otorhinolaryngology, Head and Neck Surgery, University of Erlangen-Nuremberg, 91054 Erlangen, Germany; 3 Department of Otorhinolaryngology-Head and Neck Surgery, Faculty of Medicine, Medical Center, University of Freiburg, 79106 Freiburg, Germany; 4 Department of Otolaryngology, University of Goettingen, 37075 Göttingen, Germany; 5 Department of Otolaryngology, Hannover Medical School, 30625 Hannover, Germany; 6 Department of Otolaryngology, Head and Neck Surgery, Stiftungsklinikum Proselis, Prosper-Hospital Recklinghausen, Recklinghausen, Germany; 7 Zentrum Fuer Mittelohrchirurgie (Centre for Middle Ear Surgery), ENT Practice, 44536 Luenen, Germany; 8 Department of Otorhinolaryngology, Head and Neck Surgery, University Clinic St. Poelten, 3100 St. Pölten, Austria; 9 Clinical Trials Department, Center of Hearing and Speech MEDINCUS, Kayetany, Poland; 10 Department of Teleaudiology and Screening, World Hearing Center of the Institute of Physiology and Pathology of Hearing, Kajetany, Poland; 11 Institute of Sensory Organs, Kajetany, Poland; 12 Faculty of Dental Medicine, Heart Failure and Cardiac Rehabilitation Department, Medical University of Warsaw, Warsaw, Poland; 13 World Hearing Center of the Institute of Physiology and Pathology of Hearing, Kajetany, Poland; 14 Department of Otorhinolaryngology, Medical University of Vienna, 1090 Vienna, Austria; 15 Department of Otorhinolaryngology, Head and Neck Surgery, 1030 Wiener Gesundheitsverbund, Klinik Landstraße, 1030 Vienna, Austria; 16 Department of Otorhinolaryngology, Head and Neck Surgery, Kepler University Hospital GmbH, Krankenhausstrasse 9, 4020 Linz, Austria; 17 Medical Faculty, Johannes Kepler University Linz, Altenbergerstrasse 69, 4040 Linz, Austria

**Keywords:** Ossiculoplasty, Hearing restoration, Passive middle ear implant, mCLIP partial prosthesis

## Abstract

**Purpose:**

This multicentric, retrospective study aimed to analyze the short-term safety and effectiveness of the mCLIP Partial Prosthesis**.**

**Methods:**

Patients underwent tympanoplasty with implantation of a mCLIP Partial Prosthesis. Follow-up examination included ear microscopy and pure-tone audiometry to determine the post-operative pure tone average of the frequencies 0.5, 1, 2 and 3 kHz (PTA_4_). The post-operative PTA_4_ air bone gap (ABG) was used to evaluate the audiological outcome. A post-operative minimum and maximum follow-up period was not defined. Thus, the follow-up times of each study center were different, which resulted in different follow-up times for the audiological analysis and for adverse events (AE).

**Results:**

72 (66 adults, 6 children) patients were implanted with the mCLIP Partial Prosthesis. 68 (62 adults, 6 children) patients underwent audiological examination; all 72 patients were examined for adverse events. All patients (*N* = 68): 72.1% of the patients showed a PTA_4_ ABG of ≤ 20 dB. Individual post-operative bone conduction (BC) PTA_4_ thresholds were stable in 67 patients. The mean post-operative follow-up time was 78 ± 46 days. Children (*N* = 6): 5 out of 6 children showed a PTA_4_ ABG of ≤ 20 dB. None of the children reported a BC PTA_4_ deterioration of > 10 dB HL after the implantation. The mean post-operative follow-up time was 101 ± 45 days. Adverse events (all patients, N = 72): 15 (14 adults, 1 child) patients had AEs (27 AEs and 2 Follow-Ups). The mean post-operative follow-up time was 375 days.

**Conclusion:**

Clinical data show satisfactory audiological parameters after implantation of the mCLIP Partial Prosthesis. The prosthesis is safe and effective for implantation in children and adults.

**Trial registration number:**

NCT05565339, 09 September 2022, retrospectively registered.

## Introduction

Passive middle ear implants (PMEIs) are used to reconstruct the ossicular chain to improve hearing in patients. PMEIs are designed to replace the ossicles and restore mechanical sound transmission from the tympanic membrane to the oval window. In addition to alloplastic materials, PMEIs include metals (titanium, platinum, gold), plastics (polyamide, polyethylene), Teflon®, and ceramics (hydroxyapatite, oxide ceramic, carbon, calcium phosphate ceramic, glass ceramic) [1]. Titanium is easy to implant, has a low extrusion rate and good tissue compatibility [2], and provides good functional results [3]. Titanium PMEIs have been on the market since 1994 [4], and ossicular chain reconstruction is considered the standard surgical method for hearing restoration.

**Fig. 1:**
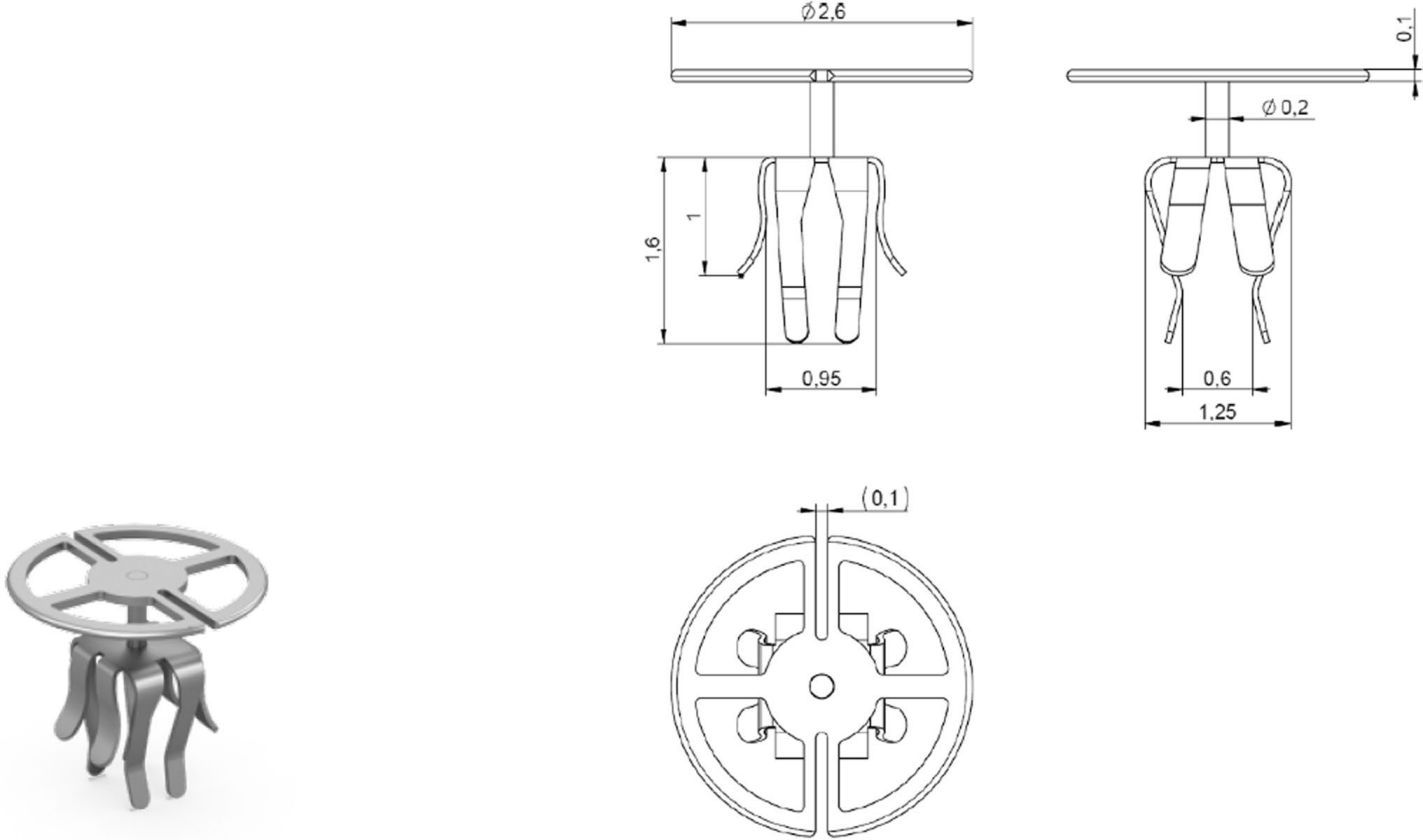
mCLIP Partial Prosthesis (left); schematic drawing of the mCLIP Partial Prosthesis (right). The titanium prosthesis consists of a contourable head plate and a standard clip. It is available in 10 different length versions

Our study provides results on patients implanted with the new mCLIP Partial Prosthesis (MED-EL, Innsbruck, Austria). The mCLIP Partial Prosthesis is made of titanium and was introduced to the market in August 2020 (Fig. 1).

## mCLIP partial prosthesis design

The mCLIP Partial Prosthesis is made of medical titanium, grade 2. The head plate has a diameter of 2.6 mm and a thickness of 0.1 mm. The mCLIP Partial Prosthesis consists of 4 longer coupling structures with a length of 1.6 mm and 4 shorter coupling structures with a length of 1 mm. For the distance between the stapes head and the tympanic membrane, called functional length, is the prosthesis available from 0.75 – 3.5 mm and determines the 10 length versions of the prosthesis: 0.75, 1.0, 1.25, 1.5, 1.75, 2.0, 2.25, 2.5, 3.0, 3.5 mm (Fig. 1).

## Material and methods

### Ethical considerations

This study was conducted in Germany, Austria and Poland (Kajetany) in agreement with the Declaration of Helsinki 2013 and was approved by the relevant German, Austrian and Polish ethics committee(s) (Wels: 1257/2022, Erlangen: 456_20 Bc, Freiburg: 22–1142-retro, Göttingen: 1/9/20, Hannover: 9456_BO_S_2020, Lünen: 2020_829_b-S, Sankt Pölten; GS1-EK-4/777–2022, Warsaw: Oświadczenie nr. 11/2021r., AKH Wien: 2296/2021, Wiener Gesundheitsverbund, Klinik Landstraße: EK_23_005_XX, Linz: 1257/2022). The study is registered at ClinicalTrials.gov under NCT05565339.

### Study design

This multicenter, retrospective follow-up study included 72 patients (72 ears), in which each patient served as his or her own control. All 72 patients were assessed for adverse events (AE); 4 of the 72 patients were excluded from the audiological analysis because of missing audiological data. For the analysis, patients implanted with the mCLIP Partial Prosthesis (regardless of implanted prosthesis length) until end of December 2022 were included.

### Audiometric methods

#### Follow-up time

Patients were evaluated pre- and post-operatively (1 pre- and 1 post-operative audiological measurement). A post-operative minimum and maximum follow-up period was not specified. Therefore, follow-up times varied among study centers, resulting in different follow-up times for audiologic analysis and AE analysis. The mean post-operative follow-up time of all patients was calculated. Post-operative PTA was calculated as a four-frequency mean of 0.5, 1, 2, and 3 kHz (PTA_4_).

#### PTA_4_ ABG

Babighian et al. defined a post-operative PTA_4_ ABG of ≤ 20 dB as successful rehabilitation [1]. The minimum success rates found in the scientific literature for a titanium partial ossicular replacement prosthesis was reported by Quesnel et al. – 53.8% of the patients achieved a PTA_4_ ABG of ≤ 20 dB post-operatively [2]. Based on [1] and [2] a post-operative PTA_4_ ABG of ≤ 20 dB by ≥ 53.8% of the patients was considered a successful outcome four our study.

#### BC PTA_4_

The individual differences (Δ) between bone conduction (BC) post-operative and pre-operative PTA_4_ were calculated to determine safety of the procedure.

#### Adverse events (AE)

All surgical-, procedure- and device-related AEs in the operated ear that occurred intra- and post-operatively were collected.

#### General information

PTA_4_ ABG and adverse events were analysed descriptively. BC PTA_4_ was calculated inferentially. Graphs were created with GraphPad Prism 7 (GraphPad Software, Inc.).

## Results

### Demographics

#### All patients (N = 72)

The 72 patients (36 female, 36 male), including 6 children, were treated in four German (Erlangen, Freiburg, Göttingen, Hannover,) and one Austrian (Wels) clinic. The mean age was 45.5 ± 18.8 years (range: 9–81 years) at the time of implantation. 34 (47.2%) patients were implanted in the left ear and 38 (52.8%) in the right ear. 47 (65.3%) patients suffered from mixed hearing loss (MHL) and 25 (34.7%) from conductive hearing loss (CHL). The current etiologies that required implantation of the mCLIP Partial Prosthesis are listed in Table 1. In 39 (54.2%) cases it was not reported whether tympanoplasty sizers were used, in 25 (34.7%) cases no tympanoplasty sizer was used, in 8 (11.1%) cases tympanoplasty sizers were used (6 × MED-EL, 2 × brand was not reported).

**Table 1:** Demographics of the patients (N = 72)

Patient number	Age at surgery [years]	Reason for PMEI implantation
1	61	Cholesteatoma / Arrosion of incus
2	51	Neuroendocrine adenoma
3	63	Cholesteatoma / Arrosion of incus
4	59	Cholesteatoma/Arrosion of incus / Malfunction of tympanic membrane
5	72	Chronic mastoiditis
6	28	Cholesteatoma
7	57	Chronic mastoiditis
8	33	Cholesteatoma
9	55	Cholesteatoma / Disruptive ossicular chain
10	63	COM / Chronic mastoiditis
11	77	Chronic mastoiditis / Otoliquorrhoe / Meningoencephalocele
12	37	Chronic mastoiditis / Disruptive ossicular chain (incudo-stapedial discontinuity) / Otitis media adhesive
13	36	Chronic otitis media
14	45	Chronic otitis media
15	32	Cholesteatoma
16	50	Chronic otitis media
17	71	Cholesteatoma
18	62	Cholesteatoma
19	53	Cholesteatoma
20	25	Fractur
21	13	Cholesteatoma
22	51	Cholesteatoma
23	14	Cholesteatoma
24	43	Cholesteatoma
25	16	Cholesteatoma
26	66	Post-traumatic incus interruption
27	56	Chronic otitis media
28	49	Otosclerosis
29	55	Cholesteatoma
30	59	Incus missing
31	57	Cholesteatoma
32	29	Dehiscence of the long process of the incus
33	70	Chronic otitis media
34	53	Cholesteatoma
35	41	Cholesteatoma
36	62	Chronic otitis media
37	26	Chronic otitis media / Cholesteatoma
38	60	Cholesteatoma
39	19	Cholesteatoma
40	21	Not reported
41	67	Dislocation of the prior prosthesis
42	60	Not reported
43	55	Not reported
44	61	Cholesteatoma
45	51	adhesive caused by arrosion of the ossicles and stapes head
46	63	Revision
47	22	Chronic otitis media
48	18	Extrusion of the prior prosthesis
49	44	Persistent otorrhea
50	26	Cholesteatoma
51	60	Chronic otitis media
52	36	Cholesteatoma
53	58	Chronic otitis media
54	29	Chronic infection of the radical cavity in childhood
55	81	Chronic otitis media / subtotal petrosectomy
56	55	Recurrent infection of the radical cavity and radical cavity cyst
57	18	Conductive hearing loss and ossicle disruption
58	50	Recurrent otorrhea
59	59	Cholesterol granuloma
60	63	Cholesteatoma
61	69	Blockage of the sound conduction after multiple ear surgeries
62	25	Cholesteatoma
63	26	Cholesteatoma
64	25	Malformation
65	11	Second look surgery after cholesteatoma
66	73	Cholesteatoma
67	13	Cholesteatoma
68	25	Congenital conductive hearing loss
69	9	Cholesteatoma / Extensive cholesteatoma of the whole mastoid and of the middle ear
70	44	COM
71	47	Adhesive
72	40	Chronic otitis media
**Mean ± SD**	**45.5 (± 18.8) years**	

COM: chronic otitis media; SD: standard deviation

## Children (N = 6):

The 6 children (3 female, 3 male) had a mean age of 12.7 ± 2.4 years (range: 9–16 years) at the time of implantation. 4 out of 6 children were implanted in the left ear and 2 in the right ear. 3 children suffered from MHL and 3 from CHL. In 2 cases it was not reported whether a tympanoplasty sizer was used, in further 2 cases no tympanoplasty sizer was used, and in other 2 cases a MED-EL tympanoplasty sizer was used.

## Audiometric results:

### PTA_4_ ABG, all patients (N = 68):

**Fig. 2:**
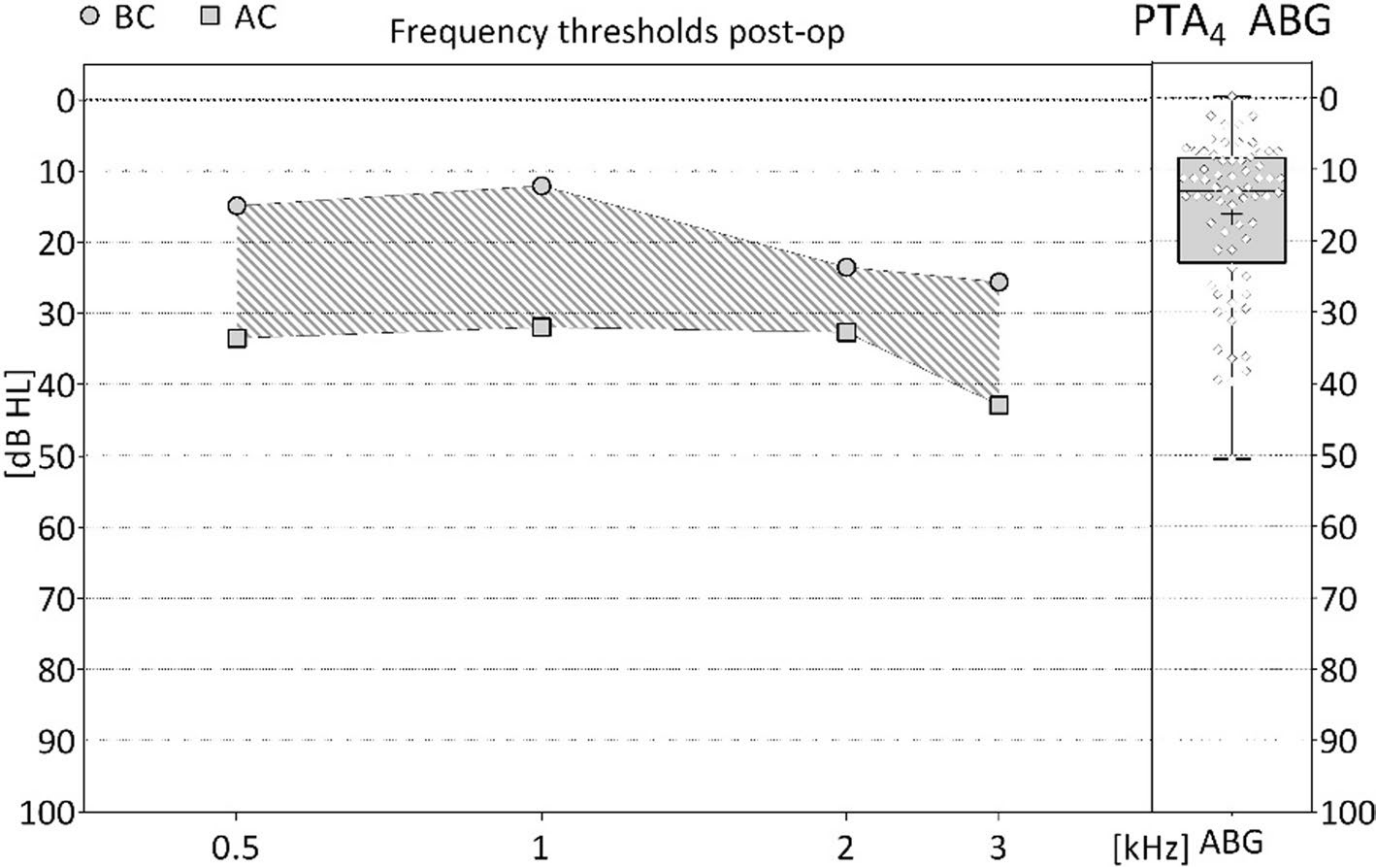
Post-operative AC and BC thresholds with the PTA_4_ ABG (N = 68). Left figure: circles = mean BC thresholds per frequency. Squares = mean AC thresholds per frequency; hatched area = ABG as difference between the results of AC and BC thresholds; mean post-operative BC PTA_4_ at 0.5/1/2/3 kHz = 14.8 ± 11.3/12.0 ± 14.3/23.4 ± 18.0/25.5 ± 18.4 dB HL. Mean post-operative AC PTA_4_ at 0.5/1/2/3 kHz = 33.4 ± 17.3/31.9 ± 18.7/32.6 ± 21.7/42.9 ± 21.9 dB HL. Right figure: PTA_4_ ABG as box plot: horizontal line = median; +  = mean; circles = distribution of individual values; whiskers = maximum and minimum. PTA_4_ ABG: mean = 16.3 ± 10.9 dB; median = 13.0 dB

The mean post-operative follow-up time was 78 ± 46 days (range: 3–302 days; median: 79 days). 49 (72.1%) of the 68 patients had a PTA_4_ ABG of ≤ 20 dB with a mean of 10.4 ± 4.4 dB (range: -0.3–19.8 dB; median: 11.0 dB) (95% CI (± e), min: 60.2%, max: 83.9%). Another 19 (27.9%) had a PTA_4_ ABG of > 20 dB, with a mean of 31.3 ± 7.5 dB (range: 21.3–50.5 dB; median: 29.5 dB) (Table 2). The mean post-operative PTA_4_ ABG of all 68 patients was 16.3 ± 10.9 dB. The first endpoint, improvement in post-operative PTA_4_ ABG of ≤ 20 dB by ≥ 53.8% of the patients was achieved. Figure 2 shows the PTA_4_ ABG results for all patients, as well as the pre- and post-operative AC and BC thresholds at the individual frequencies.

**Table 2:** Audiological data (N = 68)

Patient number	Post-operative follow-up time [days]	PTA_4_ ABG [dB]	Pre-operative / post-operative BC PTA_4_ [dB HL]
1	111	19.8	22.0/12.5
2	102	17.8	-3.8/4.0
3	20	**29.5**	11.8/10.8
5	80	**26.3**	32.5/37.5
6	89	18.8	11.3/10.0
8	120	11.3	11.3/11.3
9	79	**36.3**	12.5/15.0
11	94	17.5	**47.5/63.8**
12	121	**30.0**	1.3/1.3
13	110	11.5	20.0/9.8
14	102	15.0	22.0/17.5
15	105	11.0	6.8/10.8
16	38	8.0	15.0/12.5
17	92	6.3	20.0/11.3
18	92	**21.3**	30.0/37.5
19	14	17.5	20.0/16.3
20	92	7.5	8.8/16.3
21	113	12.5	10.0/5.0
22	118	**27.5**	13.8/13.8
23	64	**25.0**	15.0/13.8
24	83	13.8	18.8/11.3
25	68	6.3	7.5/12.5
26	10	7.5	18.8/11.3
27	61	**26.3**	22.5/22.5
28	116	3.8	15.0/13.8
29	120	**31.3**	23.8/13.8
30	118	13.8	21.3/23.8
31	103	13.8	35.0/27.5
32	88	8.8	16.3/5.0
33	80	**21.3**	46.3/45.0
34	79	7.5	17.5/18.8
35	71	10.0	27.5/11.3
36	42	8.8	27.5/26.3
37	71	**28.8**	27.5/17.5
38	87	11.3	33.8/31.3
39	91	13.8	13.8/8.8
40	89	2.5	10.0/11.3
41	101	**27.5**	38.8/42.5
42	77	6.3	30.0/20.0
43	59	3.8	35.0/25.0
44	60	11.3	56.3/46.3
45	46	13.8	10.0/18.8
46	56	7.5	22.5/22.5
47	66	**39.5**	18.5/10.5
48	21	5.8	4.5/2.8
49	21	11.3	14.3/15.5
50	3	– 0.3	30.3/36.5
51	78	12.5	26.5/15.5
52	59	13.0	12.8/11.8
53	50	**36.5**	8.5/7.0
54	41	11.3	45.3/34.3
55	45	2.5	64.3/55.5
56	21	**23.8**	12.8/14.0
57	31	7.8	6.3/3.8
58	41	13.3	10.8/9.8
59	74	8.8	12.3 / 12.8
60	57	10.3	29.0 / 25.5
61	164	**50.5**	11.0 / 8.5
62	118	**39.8**	24.3 / 28.8
63	20	14.5	11.5 / 4.3
64	38	7.0	18.0 / 8.5
65	122	13.0	7.8/6.8
66	302	10.8	38.5/29.0
67	179	14.0	22.0/19.0
68	97	8.3	32.5/18.3
69	57	9.5	7.0/5.8
71	20	**35.3**	22.5/22.5
72	20	**38.3**	62.3/65.3
**Mean ± SD**	**78 (± 46) days**	**16.3 (± 10.9) dB**	**21.4 (± 13.8) / 18.9 (± 13.9) [dB HL]**
**Median**	**79 days**	**13.0 dB**	

PTA_4_ ABG: pure tone average air bone gap at 0.5, 1, 2, 3 kHz; PTA_4_ ABG > 20 dB: bold; BC: bone conduction; BC PTA_4_ deteriorations > 10 dB HL: bold

## PTA_4_ ABG, children (< 18 years of age, N = 6):

The mean post-operative follow-up time was 101 ± 45 days (range: 57–179 days; median: 91 days). 5 out of 6 children had a PTA_4_ ABG of ≤ 20 dB (95% CI (± e), min: 36.4%, max: 100%). Patient 23 had a PTA_4_ ABG of 25.0 dB. The mean post-operative PTA_4_ ABG was 13.4 ± 6.4 dB.

## BC PTA_4_ thresholds, all patients (N = 68):

**Fig. 3:**
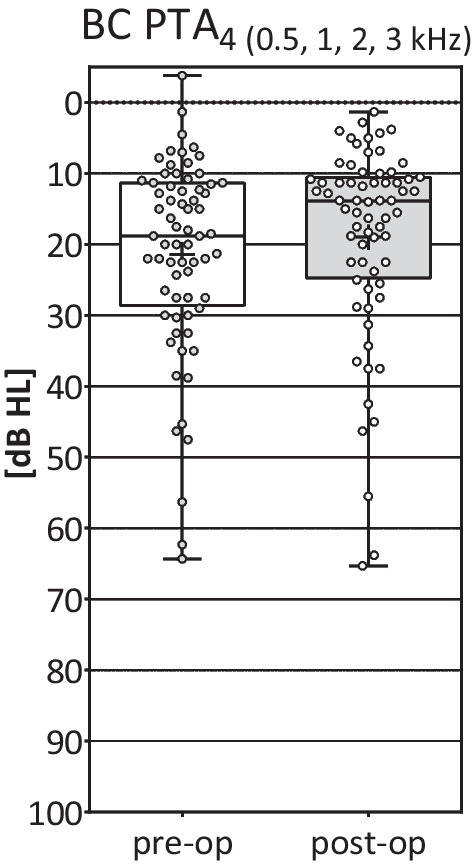
Individual differences between pre- and post-operative BC thresholds with the BC PTA_4_ (N = 68). Pre-operative outcomes were compared with post-operative outcomes. +  = mean; horizontal lines = median; circles = distribution of individual values; whiskers = maximum and minimum. BC PTA_4_ pre-/post-operative: mean = 21.4 ± 13.8/18.9 ± 13.9 dB HL

- The individual Δ BC PTA_4_thresholds were stable in 67 (98.5%) patients and within the fluctuation range of ± 5 dB HL. Patient 11 had a BC PTA_4_ deterioration of 16.3 dB HL after implantation (Table 2, Fig. 3).
- Mean BC PTA_4_ thresholds (sum of all 68 patients): The mean pre-operative BC PTA_4_threshold was 21.4 ± 13.8 dB HL (range: – 3.8–64.3 dB HL) and the mean post-operative BC PTA_4_ threshold was 18.9 ± 13.9 dB HL (range: 1.3–65.3 dB HL) (Fig. 3).

## BC PTA_4_ thresholds, children (N = 6):

- Individual BC PTA_4_ thresholds: None of the children had a BC PTA_4_ deterioration of > 10 dB HL after the implantation.
- Mean BC PTA_4_ thresholds (sum of all 6 children): The mean pre-operative BC PTA_4_ threshold was 11.5 ± 5.9 dB HL (range: 7.0–22.0 dB HL) and the mean post-operative BC PTA_4_ threshold was 10.5 ± 5.5 dB HL (range: 5.0–19.0 dB HL).

## Adverse events (AEs)

### All patients (*N* = 72)

The mean post-operative follow-up time of the 72 patients was 368 ± 177 days (range: 0–666 days; median: 375 days). 15 (20.8%) of the 72 patients had AEs (27 AEs and 2 follow-ups).

One (1.4%) underage patient showed recurrent cholesteatoma; a revision surgery was performed and the mCLIP Partial Prosthesis was replaced with a total ossicular replacement prosthesis (patient 23). Another patient (1.4%) showed a prosthesis extrusion in combination with hearing reduction (patient 24) and was treated with a hearing aid. One patient (1.4%) suffered from a fungal infection in the mastoid cavity and the headplate of the prosthesis started to migrate (patient 31) (Table 3). Due to the retrospective design of the study, there was no further information available regarding the current status of the adverse events.

**Table 3:** Adverse events

Patient number	Occurrence time after surgery [days]	Event description	Treatment
5	78	Postinflammatoric meatal fibrosis	Revision surgery of the external meatal canal
17	609	Recurrent cholesteatoma after a canal wall up tympanoplasty type III	Second look surgery was performed
19	90	Superinfected radical cavity	Suction in the radical cavity and locally treatment with Betnesol (3 × per day)
	189	Mild inflammation of the radical cavity	Suction in the radical cavity and treatment with InfectoCipro
	413	Cerumen and malodorous material	Cleaning with Otanol
22	336	Increased pressure and hearing reduction when suffering from cold symptoms	Nasal decongestant medication and treatment with Fentrinol
23	353	Recurrent cholesteatoma	Second look surgery and replacement of the current prosthesis with a total ossicular replacement prosthesis
24	20	Pain	Suction, cleaning, medication
	195	Otalgia	Keep the ear dry
	281	Wet ear	Treatment with Betnesol-N for five days
	349	Extrusion and hearing reduction (a potential device contribution could not be entirely excluded)	Hearing aid
	425	Wet ear	Treatment with Otanol for 3 days
30	NA**	Deprivation due to the long-lasting hearing loss (bad speech understanding)	Temporarily use of a hearing aid
31	5	Pain and dizziness the day before	Appointment after finishing the antibiotics
	20	Radical cavity secretion	Cleaning, touching with Otostop
	30	Radical cavity secretion	Local treatment with Aureocort ointment
	206	Ear secretion	Cleaning and treatment with Otostop
	306	Fungal infection in the radical cavity; headplate of the prosthesis migrated between 9 and 12 o’clock (a potential device contribution could not be entirely excluded)	Locally treatment with Clothrimoxazol, MRT-appointment at the end of the year
32	13	Pressure and hearing reduction	Suture removal; appointment
33	69	Wet feeling in the ear; polyp at the back part of the tympanic membrane	Suction; treatment with Otostop
35	28	Otitis externa mycotica, pain, ear secretion	Cleaning, Clotrimazol ointment, Diflucan
	82	Rusching	Hearing aid prescription
37	65	Otorrhea	Treatment with Infectociprocort
40	13	Bleeding out of the ear canal	Treatment with Diprogenta ointment strips
	152	Infection-associated seromucotympanum after a covid 19 infection	Decongestant drops
	152	Cartilage graft above the prosthesis not in the optimal position anymore medial part of the prosthesis is on the tympanic membrane; tympanic membrane has still no perforation (suspicion of protrusion of the prosthesis)	Follow-up appointment
41	101	Residual conductive hearing loss	Readjustment of the hearing device
45	4	No sense of taste in the front third of the tongue (a potential device contribution could not be entirely excluded)	Appointment; treatment with Neurobion dragees
	18	Otitis media	Treatment with Infectociprocort and Clavamox

### Children (*N* = 6)

The mean post-operative follow-up time of the 6 children was 549 ± 46 days (range: 478–606 days; median: 555 days). 1 (patient 23) out of 6 children had an AE, which is described above.

## Discussion

### History of the clip mechanism development

Hüttenbrink et al. reported in 2004, of developing a new design of prothesis, with the company Heinz Kurz GmbH, Dusslingen, Germany; this clip prosthesis consisted of elastically flexible feet, which gripped the stapes head and allowed a stable anchoring on the stapes [3]. In 2009, Hüttenbrink et al. combined the clip and the angular mechanism techniques and developed with the company Heinz Kurz GmbH, Dusslingen, Germany the new titanium angular clip prosthesis [4]. In 2011, Beutner et al. modified a common clip prosthesis type Dresden with a ball joint between the prosthesis plate and shaft [5]. The clip design of the mCLIP Partial Prosthesis is based on that of the titanium angular clip prothesis [4]. Hüttenbrink et al. reported that more contact points between the prosthesis and stapes result in a more beneficial anchoring of the prosthesis. Four (longer) of the seven clip prosthesis legs encompass the stapes head and come together between the crura; two (shorter) of the legs stabilize the prosthesis from the rear and one (shorter) leg from the front [3].

### PTA_4_ ABG

Clinical data of our study show satisfactory audiological parameters after the implantation of the mCLIP Partial Prosthesis with a mean PTA_4_ ABG of 16.3 ± 10.9 dB (*N* = 68) and stable BC PTA_4_ thresholds in 67 of the 68 patients.

During a similar trial, Gostian et al. conducted a retrospective study with 47 patients (47 ears) implanted with the Clip Partial Prosthesis Dresden Type (Heinz Kurz GmbH, Dusslingen, Germany), showing a mean post-operative PTA_4_ ABG of 20.9 ± 10.4 dB at the early follow-up at 21.1 days (range: 23.1–25.5 days). The clip design allows for stable placement and removal of the partial prosthesis without necrosis or other damage to the stapes head [7]. Our mean PTA_4_ ABG results (*N* = 68, 16.3 ± 10.9 dB) with the mCLIP Partial Prosthesis are comparable to the short-term results of Gostian et al. (20.9 ± 10.4 dB). Zaoui et al. conducted a prospective study with 52 patients implanted with a Dresden type clip partial prosthesis (Kurz, Germany). The patients were followed for 4 weeks. The mean post-operative PTA_4_ ABG was 22.4 ± 3.1 dB [8]. The mean PTA_4_ ABG of our (16.3 ± 10.9 dB) study was favourable to the results of Zaoui et al. (22.4 ± 3.1 dB) [8]. Neudert et al. reported of 29 patients implanted with a titanium clip prosthesis. The mean post-operative PTA_4_ ABG was 18.8 ± 1.6 dB. 66% of the patients had a post-operative PTA_4_ ABG of ≤ 20 dB [9]. Our mean PTA_4_ ABG results and the percentage of the mCLIP Partial Prosthesis patients which reached a PTA_4_ ABG of ≤ 20 dB is in accordance with results of Neudert et al.[9]. Omar et al. conducted a systematic review with a meta-analysis of 11 papers, which included 202 children, aged ≤ 18 years, implanted with a partial ossicular replacement prosthesis. 62.5% of the children had a post-operative ABG of ≤ 20 dB. The mean post-operative ABG ranged from 13.0 ± 6.1 to 23.8 ± 12.9 dB [10]. In our study 5 out of 6 children reached a post-operative ABG of ≤ 20 dB, with a mean range from 6.3–25.0 dB. Only 1 child had a PTA_4_ ABG of > 20 dB (patient 23, 25.0 dB). Our results are in accordance with the results of Omar et al. [10]. In sum, our audiological results are in accordance with [7–11].

### BC PTA_4_

Birk et al. evaluated the CLIP Partial FlexiBAL prosthesis (Heinz Kurz GmbH, Dusslingen, Germany) in 60 patients (62 ears). The first follow-up was in mean 19 days after the surgery. The mean BC PTA_4_ threshold was 20.2 dB HL pre-operatively and 18.1 dB HL post-operatively. The post-operative BC was stable in all 62 ears [12]. In our study the mean BC PTA_4_ threshold was for all patients (*N* = 68) 21.4 ± 13.8 dB HL pre-operatively and 18.9 ± 13.9 dB HL post-operatively, which is in accordance with the results of Birk et al. [12].

Gostian et al. reported for the early- and late follow-up that BC thresholds had no significant differences and that the mean changes in BC PTA_4_ thresholds were < 10 dB HL at all frequencies [7]. Kahue et al. reported that no patient experienced a BC PTA_4_ shift of > 15 dB HL for the short- and long-term follow-up [11]. Birk et al. [12], Gostian et al. [7], and Kahue et al. [11] included children, but they did not report separately for children. There is no consensus on comparison of post-operative sensorineural hearing loss (SNHL) [7, 11, 12]. However, when comparing the results of our study to Gostian et al. (mean changes in BC PTA_4_ thresholds were < 10 dB HL at all frequencies), and Kahue et al. (no patient experienced a BC PTA_4_ shift of > 15 dB HL), there is only one (1.5%) patient (patient 11, adult) with a BC PTA_4_ deterioration of ≥ 10 dB HL (16.3 dB HL).

### Adverse events

Three of the 72 patients (27 AEs + 2 Follow-Ups) showed 3 adverse events, which should be discussed. One patient (1.4%) showed a recurrent cholesteatoma, which resulted in the replacement of the mCLIP Partial Prosthesis with a total ossicular replacement prosthesis; this revision surgery was not device related (patient 23). One (1.4%) patient had an extrusion in combination with a hearing reduction. A hearing device was recommended or was already used after the recommendation (patient 24). One patient (1.4%) suffered from an infection, and the prosthesis started to migrate (patient 31).

Yu et al. evaluated the effectiveness and the stability of the partial ossicular replacement prosthesis using a meta-analysis. The post-operative follow-up time was < 3 years for short-term and ≥ 3 years for long-term. 3.3% of the patients had an extrusion at the short-term follow-up. 6.7% of the patients had an extrusion at the long-term follow-up. No information on the revision surgeries was given [13].

Omar et al. reported that 3% of the children had an extrusion of the prosthesis. The mean post-operative follow-up time ranged from 12–72 months [10].

In our mean follow-up period of 368 ± 177 days, it is not significant to draw a valid conclusion about the rates of revision surgeries, extrusions, and migrations. However, if our revision rate (1.4%) and our extrusion rate (1.4%) is compared to the rates of Yue et al. [13] and Omar et al. [10], they are in accordance. The rates of revision surgeries, extrusions, and migrations increase with the length of the post-operative follow-up time, which was demonstrated by the long-term safety of Yu et al. [13].

The type of partial ossicular replacement prosthesis determines the stability of the connection between the tympanic membrane and the stapes. The availability of the malleus handle, the mucosal status of the middle ear and the status of the stapes footplate, all have an important impact on the post-operative hearing results [14].

Further detrimental influences on post-operative hearing include mucosal fibrosis, drainage, revision ear surgeries and the type of surgical technique used [15]. An intra-operative and post-operative assessment of the quality of the tympanoplasty surgical technique is limited [16], and surgical interventions cannot solve every problem in the middle ear. In most cases, the mucosal function of the middle ear and the eustachian tube cannot be reconstructed completely, which results in insufficient ventilation and thus insufficient vibration of the restored middle ear [17]. In general, ossicular reconstruction depends on several anatomical factors such as aeration of the middle ear, inflammatory status of the middle ear, remnants of the ossicles, condition of the tympanic membrane (e.g., size, defect, thickness of the tympanic membrane graft), status of mucosa, experience of the surgeon and follow-up period [18], that’s why surgery results are often difficult to compare.

## Conclusion

In this short-term study, 72.1% of patients achieved a post-operative PTA_4_ ABG of ≤ 20 dB. Only one patient had a BC PTA_4_ decrease of > 10 dB HL (16.3 dB HL). The rates of performed and suggested revision surgeries, the rates of extrusions and migrations was in each case one (1.4%) patient. The follow-up time is too short to draw a final conclusion about these rates. A long-term follow-up study should be performed regarding adverse events. In summary, the mCLIP Partial Prosthesis is safe and effective for all patients (adults and children).
